# Supplementary material for: The Salmonella pathogenicity island 1-encoded small RNA InvR mediates post-transcriptional feedback control of the activator HilA in Salmonella
Source: J Bacteriol. 2025 Feb 27;207(3):e00491-24. doi: 10.1128/jb.00491-24 (PMC11925239; doi:10.1128/jb.00491-24)
Supplement: Supplemental figures and tables — Figures S1 and S2; Tables S1 to S4. [file jb.00491-24-s0001.pdf]

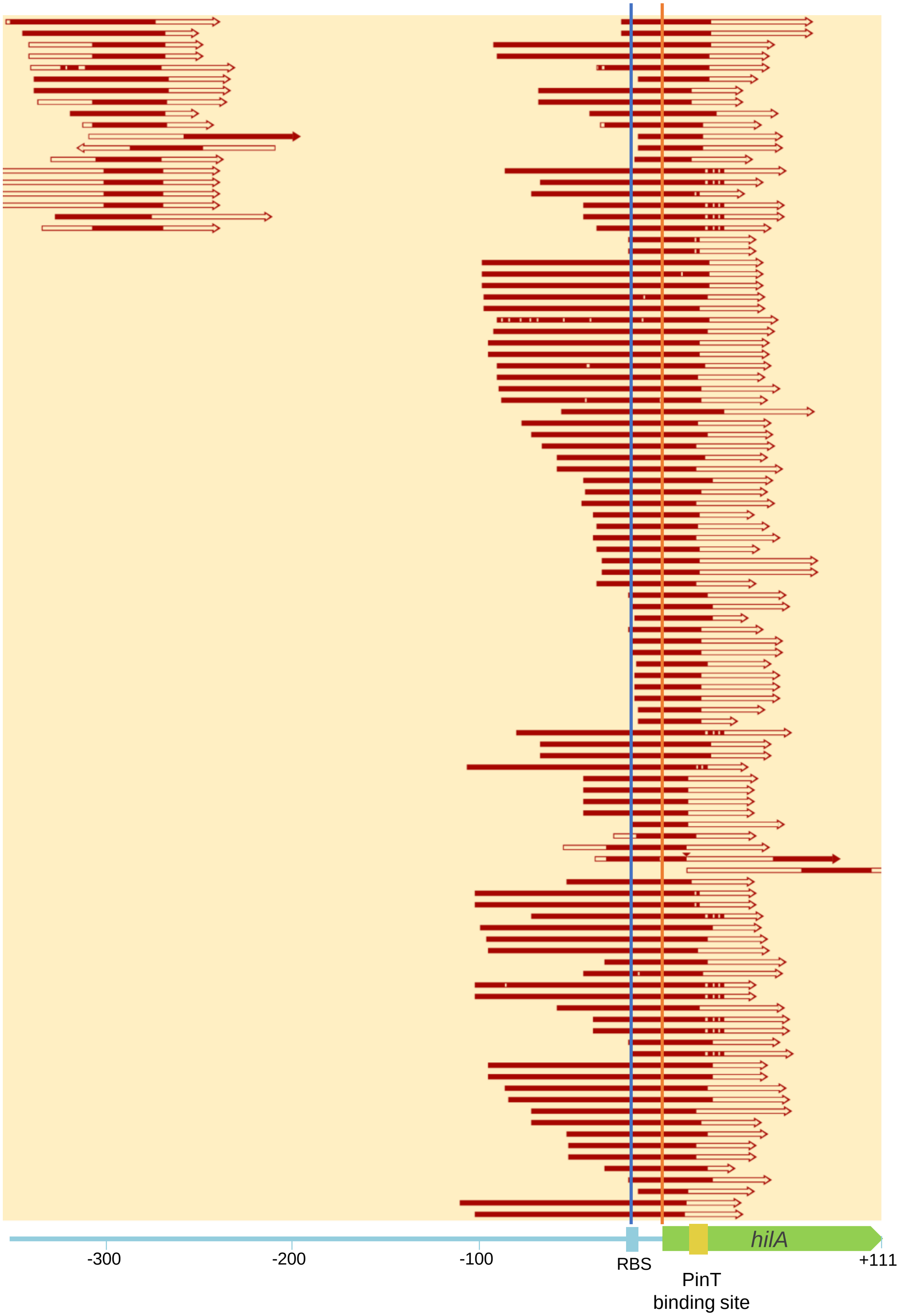

Figure S1A. rGRIL-Seq captured the direct interaction between PinT and *hilA* mRNA 5’UTR. Schematic diagram of all PinT-*hilA* chimeric reads aligned to *hilA* mRNA. Blue vertical line denotes the ribosome binding site of *hilA* mRNA. Orange vertical line denotes the start codon of *hilA* mRNA. Red solid fragment indicates *hilA* fragment in chimeric reads, red outline arrow denotes PinT fragment in chimeric reads.

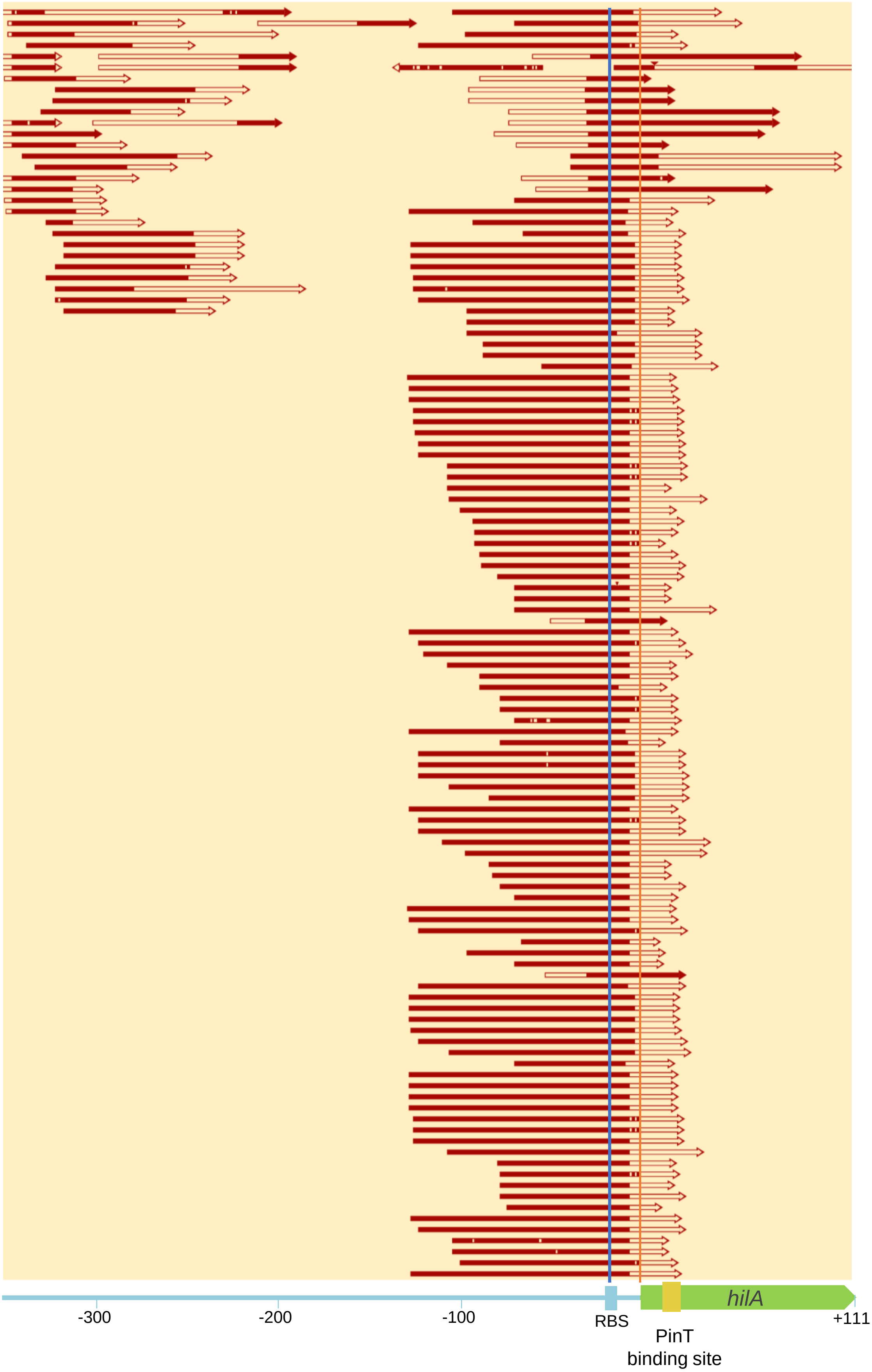

Figure S1B. rGRIL-Seq captured the direct interaction between InvR and *hilA* mRNA 5'UTR. Schematic diagram of all InvR-*hilA* chimera reads aligned to *hilA* mRNA. Blue vertical line denotes the RBS of *hilA* mRNA. Orange vertical line denotes the start codon of *hilA* mRNA. Red solid fragment indicate *hilA* fragment in chimeric reads, red outline arrow denotes InvR fragment in chimeric reads.

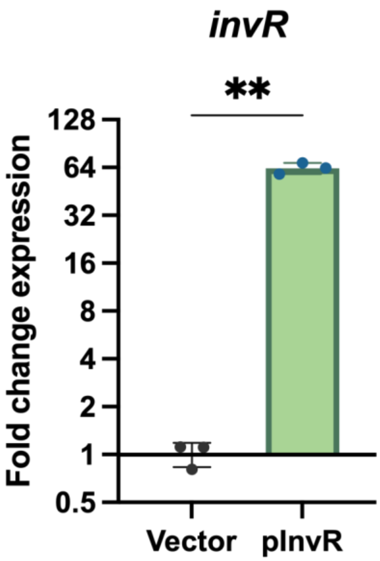

Figure S2. Real-time qPCR analysis of invR expression. *Salmonella* strains carrying vector control or pInvR expression plasmids were grown under SPI-1-inducing conditions. Fold change expression indicates the relative expression level of the target gene (*invR*) compared to the reference gene (5s RNA). Strain used: JS2333 with indicated plasmids.

Table S1. Competition assay in mice.

Strain A[a]	Strain B[a]	Infection Routed[b]	Organ[b]	Median CI[c]	*p*-value[d]	# of Mice
Δ*invR*	WT	i.p.	Sp	1.25	NS	8
		Oral	SI	0.66	NS	10
			Sp	1.78	NS	10

[a]The strains used were JS135, JS2690. [b]Bacteria were recovered from the spleen (Sp) after intraperitoneal (IP) infections or from the small intestine (SI) and spleen after oral infection. [c]The competitive index (CI) was calculated as described in Materials and Methods. [d]The Student t-test was used to compare the CIs to the inocula. NS, p >> 0.05.

Table S2. Bacterial strains used in this study.

Strain	Genotype	Deletion endpoint	Source or Reference[a]
**Salmonella**			
14028s	Wild type		ATCC[b]
JS198	LT2 *metE551 metA22 ilv452 trpB2 hisC527*(am) *galE496 xyl-404 rpsL120 flaA66 hsdL6 hsdSA29 zjg8103*::*pir*+ *recA1*		(1)
JS2333	Φ(*hilA'-'lacZ*)hyb116		(2)
JS2217	*attλ::pDX1::hilA'-lacZ* -493 to +1		(3)
JS892	Φ(*hilD'-'lacZ*)hyb139		(4)
JS2334	Φ(*rtsA'-'lacZ*)hyb6		(2)
JS248	Δ*rtsA5*		(5)
JS252	Δ*hilC113*::Cm		(5)
JS2671	Δ*hilA117*::Cm	3039761-3041761	
JS2672	Δ*hilD118*::Cm	3038103-3038965	
JS2673	Δ*ompD120*::Cm	1665155-1666238	
JS2674	Δ*invR102*::Cm	3065181-3065247	
JS2675	Φ(*rtsA'-'lacZ*)hyb6 Δ*invR102*::Cm		
JS2676	Φ(*hilC'-'lacZ*)hyb121	3033051-3032343	
JS2677	Φ(*hilC'-'lacZ*)hyb121 Δ*invR102*::Cm		
JS2117	*rne131*::Cm		(6)
JS2678	Φ(*hilA'-'lacZ*)hyb116 *rne131*::Cm		
JS2679	Φ(*invR'-lacZ*)hyb102		
JS2680	Φ(*invR'-lacZ*)hyb102 Δ*hilA117*::Cm		
JS2681	Φ(*invR'-lacZ*)hyb102 Δ*hilD118::*Cm		

JS2682	Φ(*invR'-lacZ*)hyb102 Δ*rtsA5*::Cm		
JS2683	Φ(*invR'-lacZ*)hyb102 Δ*hilC113*::Cm		
JS2684	Φ(*hilA'-'lacZ*)hyb116 Δ*invR102*::Cm		
JS2685	Φ(*hilA'-'lacZ*)hyb116 Δ*ompD120*::Cm		
JS2686	Φ(*hilA'-'lacZ*)hyb116 Δ*invR102* Δ*ompD120*::Cm		
JS2687	Φ(*hilA'-'lacZ*)hyb116 Δ*invR102* Δ*hilD118*::Cm		
JS2688	Φ(*hilD'-'lacZ*)hyb139 Δ*invR102*::Cm		
JS2689	Φ(*hilD'-'lacZ*)hyb139 Δ*ompD120*::Cm		
JS135	*zii8104*::Tn10dTc		(7)
JS2690	*zii8104*::Tn10dTc Δ*invR102*::Cm		
JS2691	Δ*hilA119*::Cm	3039745-3041754	
**E.coli**			
PM1805	MG1655 *mal::lacIq*, *ΔaraBAD araC+, lacI'::P$_{BAD}$-cat-sacB:lacZ*, mini*λtetR*		(8)
JMS6505	PM1205 *lacI'::P$_{BAD}$ -hilA'-'lacZ*		(6)
GH105	PM1805 *lacI'::P$_{BAD}$ -ompD'-'lacZ* (-68 to + 90)	1665082-1665239	
JMS6500	PM1205 *lacI'::P$_{BAD}$ -hilD'-'lacZ*		(6)
GH108	PM1805 *lacI'::P$_{BAD}$ -hilA'-'lacZ rho R66S*		
GH407	PM1805 *lacI'::P$_{BAD}$ -hilA'-'lacZ* (-285 to +30)(L1)		
GH408	PM1805 *lacI'::P$_{BAD}$ -hilA'-'lacZ* (-220 to +30)(L2)		

GH114	PM1805 *lacI'*::P_{BAD} *-hilA'*-*'lacZ* (-155 to +30)(L3)
GH409	PM1805 *lacI'*::P_{BAD} *-hilA'*-*'lacZ* (-95 to +30)(L4)
GH663	PM1805 *lacI'*::P_{BAD} *-hilA'*-*'lacZ* (-84 to +30)(L5)
GH587	PM1805 *lacI'*::P_{BAD} *-hilA'*-*'lacZ* (-60 to +30)(L6)
GH666	PM1805 *lacI'*::P_{BAD} *-hilA'*-*'lacZ* (-45 to +30)(L7)
GH667	PM1805 *lacI'*::P_{BAD} *-hilA'*-*'lacZ* (-40 to +30)(L8)
GH589	PM1805 l*acI'*::P_{BAD} *-hilA'*-*'lacZ* (-30 to +30)(L9)
GH576	PM1805 l*acI'*::P_{BAD} *-hilA'*-*'lacZ* mut1

a This study unless otherwise noted; b American Type Culture Collection

Table S3. Plasmids relevant to this study.

Plasmid	Genotype	Source or Reference
pKD46	bla PBAD*gam bet exo* pSC101 oriTS	(9)
pCP20	bla cat cI857 λPRflp pSC101 oriTS	(10)
pKD3	*bla* FRT *cat* FRT PS1 PS2 oriR6K	(9)
pKD13	*bla* FRT *kan* FRT PS1 PS4 oriR6K	(9)
pCE40	*ahp* FRT '*lacZ lacY*+ t*his* oriR6K	(1)
pKG137	*ahp* FRT *lacZY*+ t*his* oriR6K	(1)
pBRplac	AmpR, plac promoter-based expression vector	(11)
pK13-t4rnl1	pKH11-devated vector: Ptac promoter from pBTK27, CarbR, IPTG inducible, expression of T4 RNA ligase	(12)
pKH6-hilA5'UTR(rrnB terminator)	pJN105-derivated vector: P_{BAD} promoter, expression of hilA (-348 to + 111), GenR	This study
pInvR	AatII-EcoRI invR(Salmonella) containing fragment cloned into pBRplac	This study
pInvR-mut1	AatII-EcoRI invR(C32G,A31U)(Salmonella) containing fragment cloned into pBRplac	This study
pInvR-mut2	AatII-EcoRI invR(C26A,C28G)(Salmonella) containing fragment cloned into pBRplac	This study
pInvR-mut3	AatII-EcoRI invR(C26G,C28G)(Salmonella) containing fragment cloned into pBRplac	This study

pInvR-mut4	AatII-EcoRI invR(C26G,C28G,C32G)(Salmonella) containing fragment cloned into pBRplac	This study
pInvR-mut5	AatII-EcoRI invR(C26A,C28G,C32A)(Salmonella) containing fragment cloned into pBRplac	This study
pInvR-mut6	AatII-EcoRI invR(C26A,C28G,C32G)(Salmonella) containing fragment cloned into pBRplac	This study

Table S4. Oligonucleotides relevant to this study.

Description	Sequence 5'-3'
AatII-InvR-F	GACTGACGTCGTCACTTTTACGGTTGGCCATTTGT
EcoRI-InvR-R	GACTGAATTC AAAAAAAGCAGCAGCGAGGTGC
InvR-mut1-F	TTTGTCTCTTTGGTTGCATTTATCAATCTGC
InvR-mut1-R	TGGCCAACCGTAAAAGTG
InvR-mut2-F	TTACGTTGCATTTATCAATCTGCTTTTTG
InvR-mut2-R	CATACAAATGGCCAACCGTAAAAG
InvR-mut3-F	TTGCATTTATCAATCTGCTTTTTG
InvR-mut3-R	CACACAAATGGCCAACCGTAAAAG
InvR-mut4-F	TTAGGTTGCATTTATCAATCTGCTTTTTG
InvR-mut4-R	CACACAAATGGCCAACCGTAAAAG
InvR-mut5-F	TTAAGTTGCATTTATCAATCTGCTTTTTG
InvR-mut5-R	CATACAAATGGCCAACCGTAAAAG
InvR-mut6-F	TTAGGTTGCATTTATCAATCTGCTTTTTG
InvR-mut6-R	CATACAAATGGCCAACCGTAAAAG
PS1-hilAKO-F	TGCAGTAAGATAGCTACAAAACTAATCTCTATTGCTGTAGGCT GGAGCTGCTTCG
PS2-hilAKO-R	ATTACGATGATAAAAAAATAATGCATATCTCCTCTCATATGAAT ATCCTCCTTAGTTCCTATTCC
PS1-hilDKO-F	GATAATATGGAAAATGTAACCTTTGTAAGTAATAGTTGTAGGC TGGAGCTGCTTCG
PS2-hilDKO-R	TTAATGGTTCGCCATTTTTATGAATGTCGATGGCGTCATATGA ATATCCTCCTTAG
PS4-hilCKO-F	TAAAGGCAGTTTGCGAATTGAAAACGAATTTGGGGAGTTCATT CCGGGGATCCGTCGACC
PS1-hilCKO-R	TCAATGGTTCATTGTACGCATAAAGCTAAGCGGTGTAATCTGT AGGCTGGAGCTGCTTCG
PS1-ompDKO-F	ACACGCTAAGAAAATTATAAGGATTATTAAAATGATGTAGGCT GGAGCTGCTTCG
PS2-ompDKO-R	GAAAGGACTGGCTTTGTATTCAGACTACAACAAAACATATGAA TATCCTCCTTAGTTCCTATTCC
PS2-invRKO-F	ATATAATTATTATAGCTATGGTCACTTTTACGGTTGGCCACATA TGAATATCCTCCTTAGTTCCTATTCC
PS1-invRKO-R	TTCCCAAGTCTGGGAGGCCGTTCTTTATCACAAATTGTAGGCT GGAGCTGCTTCG

hilA-L1	TCGCAACTCTCTACTGTTTCTCCATACTCTCTCTGCACCAGGAT ATACGGCAGCGTCCATTCGATAATCACAGTTAGTTATAACAATA TTATTACCAACATGTCAGTTATTTAAAGCACAGGCATAAGCTAA ATAATCAAATGTTAAAAACATATAAACCCGAGCCCGTAGAATAT GACATTAAGCTCATAATAAAAGCTCAACCTGACCGTTAGTACTA ACAGCAGAATTACTGAAACAGTAGATTCTATCCTAACGACTTGT ATTAGTTATTATAACTTTTCACCCTGTAAGAGAATACACTATTAT CATGCCACATTTTAATCCTGTTCCTGTATCGGTCGTTTTACAAC GTCGTGACTGGG
hilA-L2	TCGCAACTCTCTACTGTTTCTCCATATTACCAACATGTCAGTTA TTTAAAGCACAGGCATAAGCTAAATAATCAAATGTTAAAAACAT ATAAACCCGAGCCCGTAGAATATGACATTAAGCTCATAATAAAA GCTCAACCTGACCGTTAGTACTAACAGCAGAATTACTGAAACA GTAGATTCTATCCTAACGACTTGTATTAGTTATTATAACTTTTCA CCCTGTAAGAGAATACACTATTATCATGCCACATTTTAATCCTG TTCCTGTATCGGTCGTTTTACAACGTCGTGACTGGG
hilA-L3	TCGCAACTCTCTACTGTTTCTCCATAAACCCGAGCCCGTAGAA TATGACATTAAGCTCATAATAAAAGCTCAACCTGACCGTTAGTA CTAACAGCAGAATTACTGAAACAGTAGATTCTATCCTAACGACT TGTATTAGTTATTATAACTTTTCACCCTGTAAGAGAATACACTAT TATCATGCCACATTTTAATCCTGTTCCTGTATCGGTCGTTTTAC AACGTCGTGACTGGG
hilA-L4	TCGCAACTCTCTACTGTTTCTCCATTACTAACAGCAGAATTACT GAAACAGTAGATTCTATCCTAACGACTTGTATTAGTTATTATAA CTTTTCACCCTGTAAGAGAATACACTATTATCATGCCACATTTT AATCCTGTTCCTGTATCGGTCGTTTTACAACGTCGTGACTGGG
hilA-L5-F	ACGCTTTTTATCGCAACTCTCTACTGTTTCTCCAT GAATTACTGAAACAGTAGATT
hilA-L6	TCGCAACTCTCTACTGTTTCTCCATTCCTAACGACTTGTATTAG TTATTATAACTTTTCACCCTGTAAGAGAATACACTATTATCATGC CACATTTTAATCCTGTTCCTGTATCGGTCGTTTTACAACGTCGT GACTGGG
hilA-L7	TCGCAACTCTCTACTGTTTCTCCATTTAGTTATTATAACTTTTCA CCCTGTAAGAGAATACACTATTATCATGCCACATTTTAATCCTG TTCCTGTATCGGTCGTTTTACAACGTCGTGACTGGG
hilA-L8-F	ACGCTTTTTATCGCAACTCTCTACTGTTTCTCCAT TATTATAACTTTTCACCCTGTAAGA

hilA-S	TCGCAACTCTCTACTGTTTCTCCATTTTCACCCTGTAAGAGAATACACTATTATCATGCCACATTTTAATCCTGTTCCTGTATCGGTCGTTTTACAACGTCGTGACTGGG
hilA-lacZ-R	TAACGCCAGGGTTTTCCCAGTCACGACGTTGTAAAACGACCGATACAGGAACAGGATTAA
hilA-mut1	TCGCAACTCTCTACTGTTTCTCCATACAAAACTAATCTCTATTGCAATGAGGCCAAGTTAAATATGTAAATATTTAGATGCCCGGCGCTGACTCTCTCTGCACCAGGATATACGGCAGCGTCCATTCGATAATCACAGTTAGTTATAACAATATTATTACCAACATGTCAGTTATTTAAAGCACAGGCATAAGCTAAATAATCAAATGTTAAAAACATATAAACCCGAGCCCGTAGAATATGACATTAAGCTCATAATAAAAGCTCAACCTGACCGTTAGTACTAACAGCAGAATTACTGAAACAGTAGATTCTATCCTAACGACTTGTATTAGTTATTATAACTTTTCACCCTCAAAGAGAATACACTATTATCGTCGTTTTACAACGTCGTGACTGGG

REFERENCES

1. Ellermeier CD, Janakiraman A, Slauch JM. 2002. Construction of targeted single copy lac fusions using λ Red and FLP-mediated site-specific recombination in bacteria,

Gene. Gene 290:153-161.

2. Kim K, Palmer AD, Vanderpool CK, Slauch JM. 2019. The Small RNA PinT Contributes to PhoP-Mediated Regulation of the Salmonella Pathogenicity Island 1 Type III Secretion System in Salmonella enterica Serovar Typhimurium. J Bacteriol 201.
3. Palmer AD, Kim K, Slauch JM. 2019. PhoP-Mediated Repression of the SPI1 Type 3 Secretion System in Salmonella enterica Serovar Typhimurium. J Bacteriol 201.
4. Cott Chubiz JE, Golubeva YA, Lin D, Miller LD, Slauch JM. 2010. FliZ regulates expression of the Salmonella pathogenicity island 1 invasion locus by controlling HilD protein activity in Salmonella enterica serovar typhimurium. J Bacteriol 192:6261-70.
5. Ellermeier CD, Slauch JM. 2003. RtsA and RtsB coordinately regulate expression of the invasion and flagellar genes in Salmonella enterica serovar Typhimurium. J Bacteriol 185:5096-108.
6. Kim K, Golubeva YA, Vanderpool CK, Slauch JM. 2019. Oxygen-dependent regulation of SPI1 type three secretion system by small RNAs in Salmonella enterica serovar Typhimurium. Mol Microbiol 111:570-587.
7. Stanley TL, Ellermeier CD, Slauch JM. 2000. Tissue-Specific Gene Expression Identifies a Gene in the Lysogenic Phage Gifsy-1 That Affects <i>Salmonella enterica</i> Serovar Typhimurium Survival in Peyer's Patches. Journal of Bacteriology 182:4406-4413.
8. Lee HJ, Gottesman S. 2016. sRNA roles in regulating transcriptional regulators: Lrp and SoxS regulation by sRNAs. Nucleic Acids Res 44:6907-23.
9. Datsenko KA, Wanner BL. 2000. One-step inactivation of chromosomal genes in Escherichia coli K-12 using PCR products. Proceedings of the National Academy of Sciences 97:6640-6645.
10. Cherepanov PP, Wackernagel W. 1995. Gene disruption in Escherichia coli: TcR and KmR cassettes with the option of Flp-catalyzed excision of the antibiotic-resistance determinant. Gene 158:9-14.
11. Guillier M, Gottesman S. 2006. Remodelling of the Escherichia coli outer membrane by two small regulatory RNAs. Mol Microbiol 59:231-47.
12. Han K, Tjaden B, Lory S. 2016. GRIL-seq provides a method for identifying direct targets of bacterial small regulatory RNA by in vivo proximity ligation. Nature Microbiology 2:16239.
